# The measurement of Central Corneal Thickness

**DOI:** 10.22336/rjo.2023.29

**Published:** 2023

**Authors:** Georgiana Camburu, Mihail Zemba, Călin Petru Tătaru, Victor Lorin Purcărea

**Affiliations:** * “Carol Davila” University of Medicine and Pharmacy, Bucharest, Romania

**Keywords:** Central Corneal Thickness, OCT, Pachymetry, Specular Microscopy

## Abstract

We aimed to compare five different devices that measure Central Corneal Thickness. The Central Corneal Thickness (CCT) is an important parameter in ophthalmology. It is involved in the management of various eye conditions such as: glaucoma, keratoconus, contact lens wearing, corneal dystrophies, refractive surgery and keratoplasty. We measured the CCT using OCT, Topographer (TOPO), Ultrasonography Pachymeter (US), Specular Microscope (MS), and Non-contact Tonometer (TONO). In the analysis of the data collected from 59 patients we found the following mean values: US - 554.51 ± 29.849 μm, OCT - 548.73 ± 31.080 μm, TOPO - 553.76 ± 29.845 μm, MS - 564 ± 32.637 μm, and TONO - 538.9 ± 35.657 μm. Our results confirmed the strong correlation between techniques.

**Abbreviations: **OCT = Optical Coherence Tomography, CCT = Central Corneal Thickness, TOPO = Topographer, US = Ultrasonography Pachymeter, MS = Specular Microscope, TONO = Non-contact Tonometer

## Introduction

The Central Corneal Thickness is an important parameter in ophthalmology. It is involved in the management of various eye diseases such as: glaucoma, keratoconus, contact lens wearing, corneal dystrophies and patients about to undergo surgery, meaning refractive surgery and performing keratoplasty. Determining the Central Corneal Thickness is an essential factor in establishing the diagnosis of glaucoma. The value of the intraocular pressure of patients whose corneal thickness exceeds 540 μm will be false positive, and artificially increased. Moreover, the intraocular pressure of patients whose pachymetry will be below this value, will be shown below the real value [**1**,**2**].

Pachymetry values are important in the selection of patients for refractive surgery. Pachymetry can detect subclinical forms of pathologies such as Keratoconus, Pellucid Marginal Corneal Degeneration, Fuchs Dystrophy [**3**].

Pachymetry is an indicator of corneal health, but the value varies in normal patients. The thinnest part is usually located 1.5 mm temporally from the corneal center. The corneal thickness varies between 420 and 625 μm. The average thickness is 515 μm in the center of the cornea. In the paracentral region, the thickness varies from 522 μm lower to 574 μm upper. In the peripheral area, the thickness is 633 μm lower and 673 μm upper [**4**].

Paracentral and peripheral measurements tend to become thinner with age, but this trend has not been statistically significant. The central region (4 mm from the optic area) is usually thinner than the middle of the corneal periphery (4-9 mm from the optic area), which is in turn thinner than the corneal periphery (9 mm from the optic area). Therefore, endothelial dysfunction is suspected in case of a cornea greater than the peripheral Central Corneal Thickness. Corneal thickness is an indirect index of the pump function of the corneal endothelium, which can be affected by intraocular pressure. As a pathological variant, there is also the possibility of corneal thinning in Keratoconus or marginal pellucid degeneration, but also its thickening as endothelial dysfunction, like Fuchs dystrophy. If intraocular pressure is normal, epithelial edema develops when the thickness of the stroma is increased by 40% and the central corneal thickness becomes 700 μm. If the increase in thickness is only 20%, or the corneal pachymetry is 620 μm, the risk of corneal decompensation is high. The other uses of pachymetry include the functional status of the corneal transplant and the evaluation of keratoconus progression. In contact lens wear, corneal edema and hypoxia may occur in daily, extensive, or therapeutic use. The increase in corneal thickness varies by 4% during blinking, 10% in extended contact lens wear, and 1% during sleep. On biomicroscopic examination, the corneal striae become visible at 8% increase in thickness, folds at 12%, and loss of transparency occurs at an increase of 20%. High altitude can also generate a significant increase in corneal thickness, usually due to endothelial dysfunction. Techniques for measuring central corneal thickness (CCT) include optical pachymetry, ultrasound pachymetry, confocal microscopy, optical light ray passage analysis, topography, and optical coherence tomography. Optical methods for determining CCT consider the image projected onto the tear film or anterior corneal surface [**1**,**5**].

We aimed to compare different ophthalmological devices in measuring corneal thickness. The type of study chosen was cross-sectional, obtaining information from a sample of patients at a given time. 

## Patients

A comparative study was conducted, involving 59 randomized patients who presented to the Ophthalmology Department during 2017-2020. Patients underwent a complete ophthalmologic examination. The first step was the anamnesis, in which a history of pathology or ophthalmologic intervention, administered treatments and systemic pathologies that could influence the measurements performed were excluded. We looked for factors that could become sources of error later. The measurement of visual acuity with and without correction was performed using the Snellen Test at 6 m, according to data taken from the auto kerato-refractometer. During the biomicroscopic and fundus examination, and annexes of the eyeball, it was observed that patients had various refractive errors (myopia, hyperopia, forms of astigmatism), but classified as low and medium values. Informed consent was taken from all the patients after the protocol and possible risks were explained.

## Materials and methods

- AS-OCT; 

- Corneal topography;

- Corneal ultrasonography; 

- Specular microscopy; 

- Non-contact tonometry. 

Statistical data processing and analysis were performed with IBM Statistical Package for the Social Sciences version 25, Microsoft Office Excel version 16.32 and Microsoft Word version of the same software package.

The genders in the studied group were: 60% women and 40% men, the frequencies being 36 and 23.

*Ultrasonography method (US)*

Using this method, it was observed that the number of measurements analyzed was of 59 patients, with an average of 554.51. The range of measurements was delimited by values between 488 and 612, with the value 551 as the median. The dispersion of the obtained results could be quantified by calculating the standard deviation of 29.849, the skewness and kurtosis of values being -0.185, 0.067. The confidence interval of 95% was chosen, with the lower limit of 540.92 and the upper limit of 557.05. The next step was to apply the Kolmogorov-Smirnov and Shapiro-Wilk tests to certify the normal distribution of results obtained in the studied sample. Obtaining the values 0.200 and 0.338, well above the value 0.05 set as a threshold, would have generated the rejection of the null hypothesis, thus considering the sample normally distributed.

*Optical Coherence Tomography (OCT) method*

The first step in the data analysis was to process it to define the ends of the measured range. The minimum was 487 and the maximum was 548.73 μm corneal thickness. The mean and median values were 548.73 and 550. The dispersion was 31.080, skewness - 0.321 and kurtosis 0.096. The next step to outline the image of the studied group was to describe the distribution normality. Obtaining the value of 0.200 and 0.605 achieved the rejection of the null hypothesis, thus considering the sample normally distributed.

*Topography Method (TOPO)*

During the analysis of the data we observed that the average and median almost corresponded, a sign of the symmetry of values around median. This was not surprising as the skewness value of 0.122 was obtained, while the standard deviation was 29.845. The minimum and maximum range of measurements was 496 and 622. The confidence interval set for the 95% value was limited to a thickness of 537 and 552.76. The test with the Kolmogorov-Smirnov and Shapiro-Wilk algorithms was necessary to describe the normality of distribution of the measurements obtained with the topographer. These tests quantified the distribution as normal. 

*Specular Microscopy (MS) Method*

The Specular Microscopy method found measurements ranging from 491 to 626. The median was 568 and the mean was 564. The standard deviation found was 32.637. The skewness and kurtosis of the lot presented values of -0.236 and -0.209. The 95% confidence interval was set between 555.85 and 572.86. After applying the Kolmogorov-Smirnov and Shapiro-Wilk tests, the normal distribution of the batch was demonstrated.

*Non-contact tonometry method*

The first stage consisted in analyzing the indicators of descriptive statistics: the median being 538, close to the value of the mean, 538.9 suggesting a strong symmetry of the sample. The standard deviation with a sign of dispersion was 35.657. The range of measurements using this technique was between 467-620. Skewness and kurtosis were -0.430 and 0.09. The 95% confidence interval was between 529.61-548.19. Kolmogorov-Smirnov and Shapiro-Wilk tests were used to determine the normal distribution, a fundamental step in the subsequent statistical processing of data.

Subsequently, ANOVA testing was also chosen to statistically test the differences between the groups obtained by measuring corneal thicknesses with the 5 methods.

The results we outlined were around the Bland Alman Charts. These are statistical tools used to identify differences found in determining a parameter measured by 2 techniques applied to the same patient. The graphs provided the opportunity to detect measurements (of patients) that did not fall within the agreement limits.

**Graph 1:**
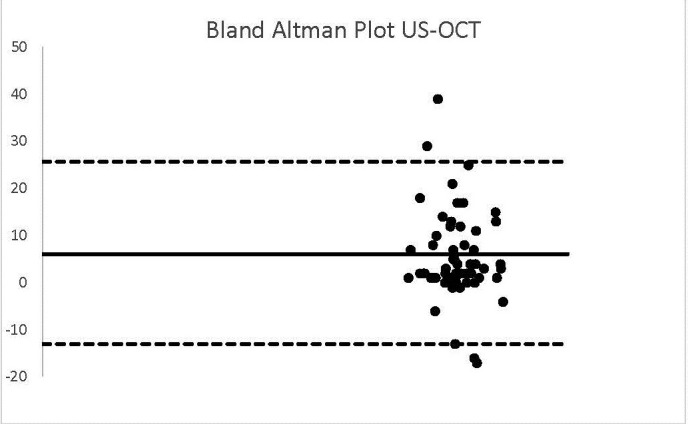
US-OCT

In the case of analyzing the data resulting from the US and OCT technique, a bias equal to the value of 6 was obtained. The standard deviation was 9.84 and the agreement interval was outlined between -13.51 and 25. It was observed that 4 patients did not fit into this range.

**Graph 2:**
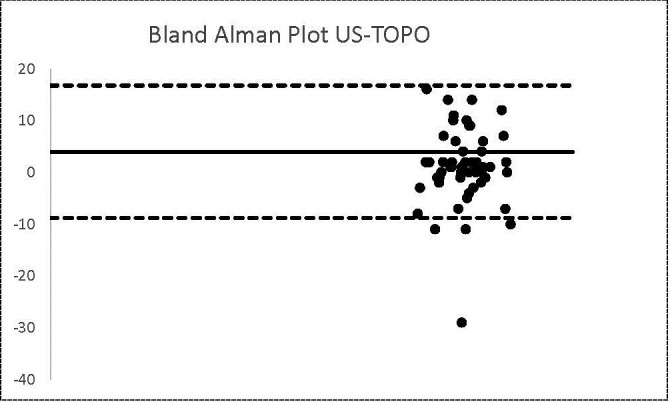
US-TOPO

The same number of patients were found in the case of US-TOPO methods. The calculated bias was 1 and the agreement limits were -8.82 and 16.75.

**Graph 3:**
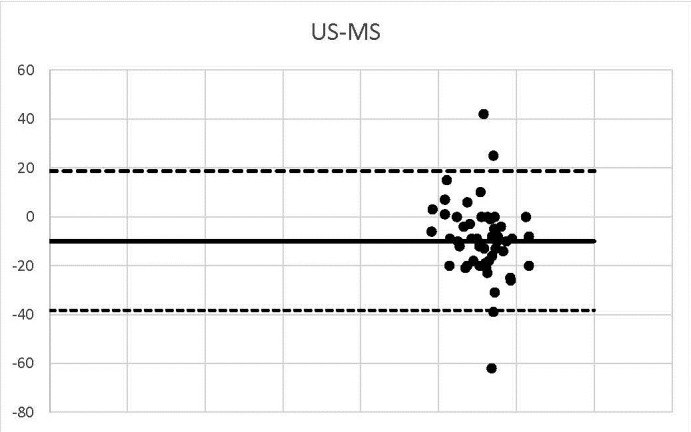
US-MS

3 corneal thickness measurements detected using US-MS did not match the range of 95% agreement.

**Graph 4:**
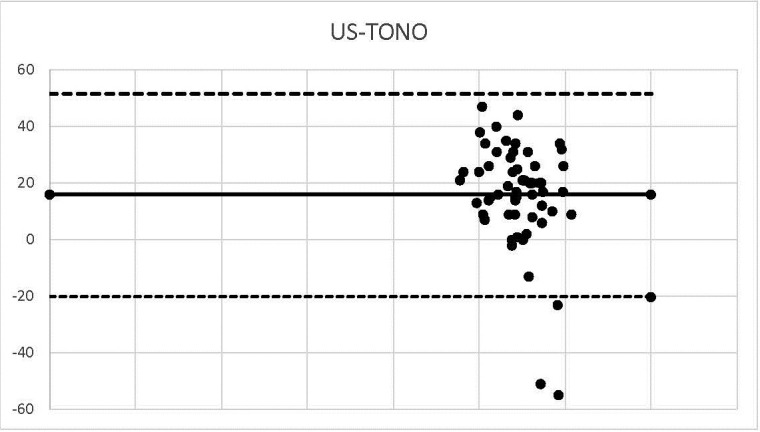
US-TONO

3 corneal thickness values were within the agreement range (-38.34, 18.646).

**Graph 5:**
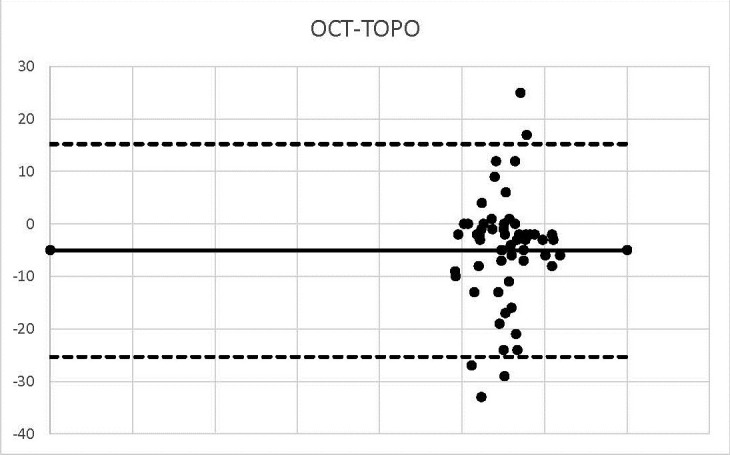
OCT-TOPO

The bias calculated in the case of OCT-TOPO was -5, so the values were shaped around this value.

**Graph 6:**
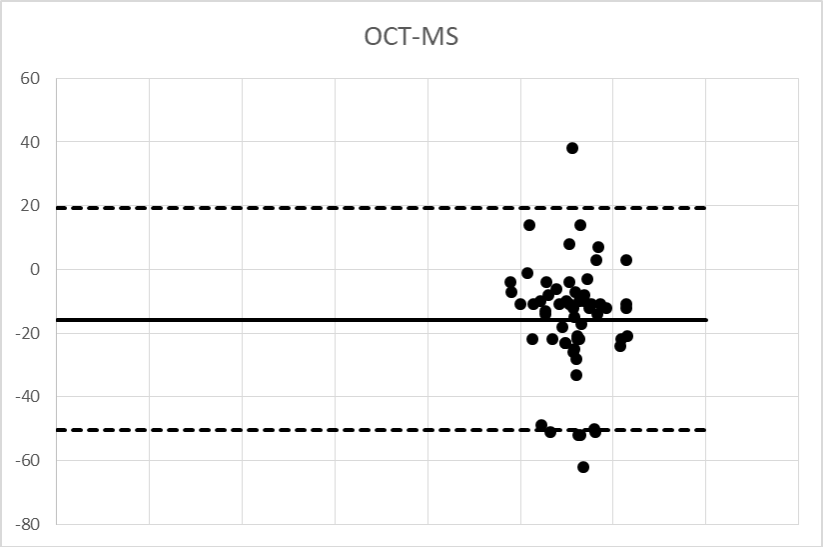
OCT-MS

2 values were outside the range -50.4 and 19.14.

**Graph 7:**
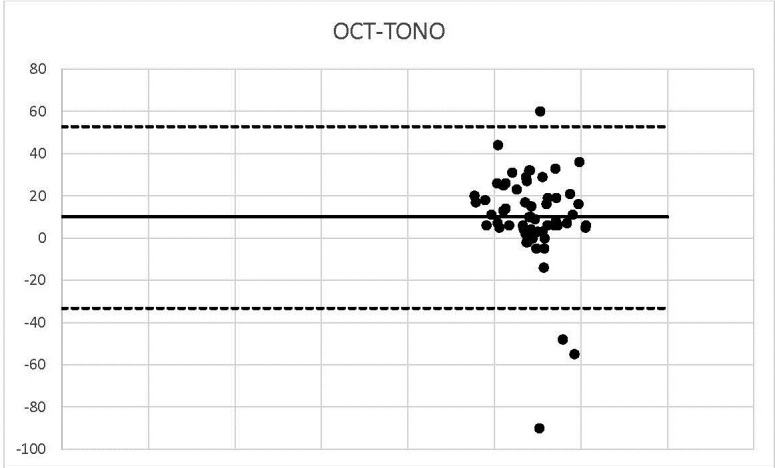
OCT-TONO

**Graph 8:**
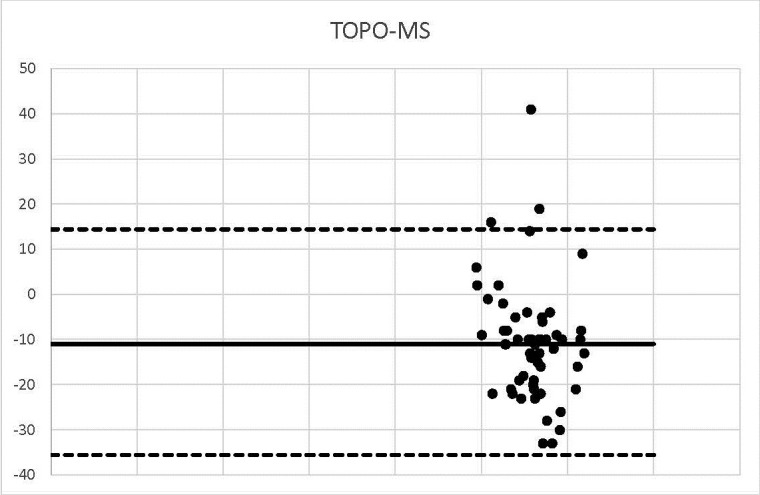
TOPO-MS

Three measurements were identified outside the agreement range.

**Graph 9:**
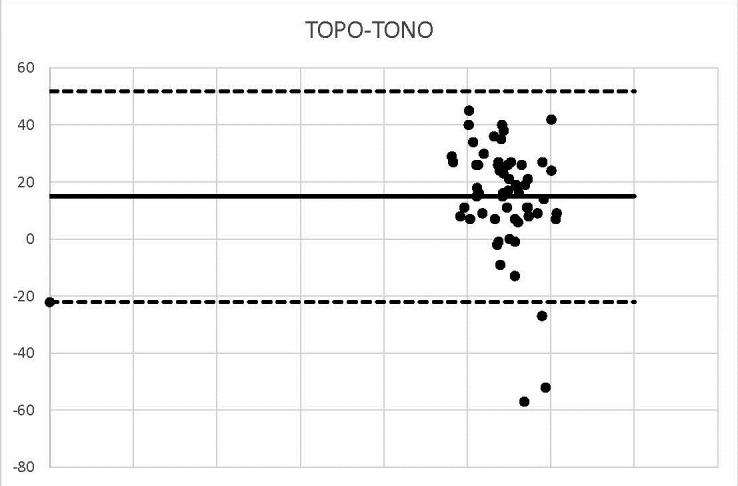
TOPO-TONO

**Graph 10:**
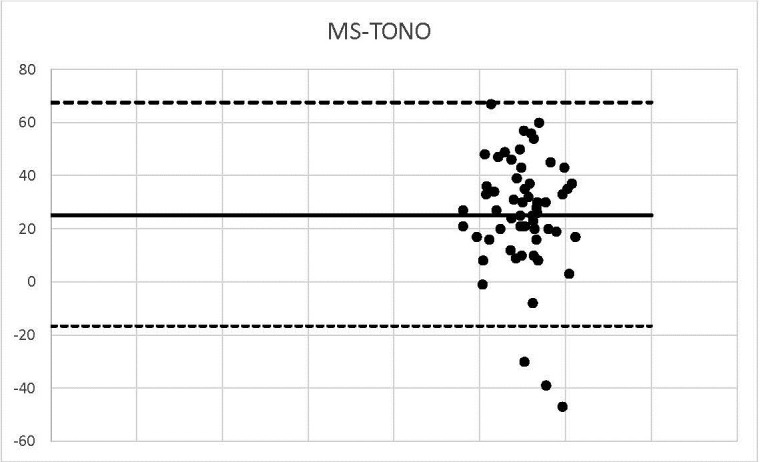
MS-TONO

In the comparative analysis of the results obtained by measuring corneal thicknesses with various techniques, we also aimed to find out the correlation between the values of different ophthalmologic devices. In the previous stage, we established the normality of distribution of the 5 technics studied. Furthermore, it was necessary to apply the Pearson type correlation. The calculated correlation between US and OCT was 0.949. Statistically, the value exceeding >0.5 was considered to reveal a strong correlation. Values around a maximum of 1 signified a very strong intensity of the relationship between the two methods of determination. The Pearson value between US and TOPO was 0.971, which demonstrated a strong correlation. The correlation between US and MS methods was determined to be 0.896. The correlation between US and TONO was 0.856. The Pearson value between OCT and TOPO was 0.942. The correlation between OCT and MS was a strong one, of 0.843. Testing the correlation between OCT and TONO established the close connection between the 2 techniques, P=0.788. The correlation between TOPO and MS was 91.9%. The Pearson value studied was 0.846. TONO and MS had a correlation of 80%.

## Discussions

The first stage consisted in analyzing the indicators of descriptive statistics. The next step was to apply the Kolmogorov-Smirnov and Shapiro-Wilk tests to certify the normal distribution of results obtained in the studied sample. The values obtained were 0.200 and 0.338, 0.200 and 0.605, 0.200, 0.200, well above the value 0.05 set as a threshold, which generated the rejection of the null hypothesis, this way considering the samples normally distributed. 

The average measurement obtained by the US technique was 554.5129.849, and by OCT 548.7331.0, the difference between these averages being 5.78, in favor of the US. The average of the values obtained using the topography method was 553.76 to 29.845. Values higher than 0.75 were observed in the case of the ultrasound method. Regarding corneal thickness, the specular microscopy technique provided values averaging 564.3632.637, the difference being 9.85, with values increased by using ultrasound.

The mean difference between the two US and TONO investigations was 15.61, the mean non-contact tonometry method being 538.90 ± 35.657. The comparison of averages between OCT and TOPO was by difference -5.03, with averages between 548.7531 and 553.76 to 29.845. The values obtained by the MS method averaged, being of 564.36, 32.637, with a difference of -5.63. The difference in averages between the 2 methods was 9.83. 

An increase in the mean MS values was observed by 10.6 compared to the mean TOPO. A difference of 14.86 was observed between the averages of TOPO and TONO techniques. The difference between MS and TONO averages was 25.46. In the comparative analysis of the results obtained by measuring corneal thicknesses with various techniques, the calculated correlation between US and OCT was 0.949. Statistically, the value exceeding >0.5 was considered to reveal a strong correlation. Values around a maximum of 1 denoted the very strong intensity of the relationship between the two methods of determination. The value of the Pearson Index between US and TOPO was 0.971, which demonstrated a strong correlation. The correlation between US and MS methods was determined to be 0.896. The correlation between US and TONO was 0.856. The Pearson value between OCT and TOPO was 0.942. The correlation between OCT and MS was strong, of 0.843. Testing the correlation between OCT and TONO established the close connection between the 2 techniques, P=0.788. The correlation between TOPO and MS was 91.9% and the Pearson value studied was 0.846. TOPO-TONO TONO and MS techniques had a correlation of 80%.

We outlined the results around the Bland Alman Charts. These are statistical tools used to identify differences found in determining a parameter measured by 2 techniques applied to the same patient. The graphs provided the opportunity to detect measurements (of patients) that did not fall within the agreement limits. The mean values that were not found in the 95% agreement range between two methods were 3 in the case of US-OCT, US-Ms, US-TONO, OCT-MS, TOPO-MS, TOPO-TONO, MS-TONO. Two couples, US-OCT and US-TOPO, scored an average of 4, and OCT-TOPO an average of 5. These results confirmed the strong correlation between the techniques. 

## Conclusions

The average measurement obtained by the US technique was 554.5129.849 and by OCT 548.73 ± 31.0. The difference between these averages was 5.78, in favor of the US. The average of the values obtained using the topography method was 553.76 ± 29.845. In measuring corneal thickness, the specular microscopy technique provided values averaging 564.36 ± 32.637. The difference was 9.85, with values increased by using MS. The difference in averages between the two US and TONO investigations was 15.61 and the mean of the non-contact tonometry method 538.90 ± 35.657. Thus, we could argue that the values resulting from TOPO were values close to those of the US. The increased variation between US and MS media might have been due to the principle of operation of specular microscopy. It is known from literature that measurements up to 30 μm higher can be obtained. We observed an underestimation of corneal thickness with the TONO technique.

The comparison of averages between OCT and TOPO was by difference -5.03 with averages 548.75 ± 31 and 553.76 ± 29.845. The values obtained by the MS method averaged 564.36 ± 32.637, with a difference of -5.63. The mean difference between the 2 methods was 9.83 TONO-OCT. An increase in the mean MS values was observed by 10.6 compared to the mean TOPO. A difference of 14.86 was observed between the averages of TOPO and TONO techniques. The difference between MS and TONO averages was 25.46.

The scientific research objectives initially proposed when designing the research were met, a strong correlation being observed between the five types of devices used in corneal thickness measurement. Given the dynamism of medical technology and the application of improvements to the current operating principles, improvements that can be based on the results from the research, measurements with better accuracy will be obtained.

**Conflict of Interest statement**

The authors state no conflict of interest.

**Informed Consent and Human and Animal Rights statement**

Informed consent has been obtained from all individuals included in this study.

**Authorization for the use of human subjects**

Ethical approval: The research related to human use complies with all the relevant national regulations, institutional policies, is in accordance with the tenets of the Helsinki Declaration, and has been approved by the ethical committee “Carol Davila” University of Medicine and Pharmacy, Bucharest, Romania.

**Acknowledgements**

None.

**Sources of Funding**

This article was published with the support granted by the project entitled “Net4SCIENCE: Applied doctoral and postdoctoral research network in the fields of smart specialization Health and Bioeconomy”, project code POCU/993/6/13/154722.

**Disclosures**

None.
